# Olfactory Nerve Schwannoma: A Case Report and Review of the Literature

**DOI:** 10.1055/s-0038-1669991

**Published:** 2018-09-10

**Authors:** Mahmoud M. Taha, Amr AlBakry, Magdy ElSheikh, Tarek H. AbdelBary

**Affiliations:** 1 Department of Neurosurgery, Zagazig University, Zagazig, Egypt

**Keywords:** olfactory nerve Schwannoma, anterior cranial fossa schwannoma, olfactory groove schwannoma, olfactory, schwannoma

## Abstract

Schwannomas are benign tumors, which arise from the Schwann cells of the central or peripheral nerves. They form 8% of all intracranial tumors and most of the cases arise from vestibular division of the 8 ^th^ cranial nerve. Rare cases are shown to arise from the olfactory or optic nerve, being devoid of myelin sheath. Up to date and according to our best of knowledge, 66 cases have been reported till now. Here we present a review of the literature and a case report of a 56-year-old male with an accidently discovered anterior cranial fossa schwannoma, following a road traffic accident. Tumor was completely excised, using a right frontal approach. Histopathology revealed Antoni-A cellular pattern. Although rare, but olfactory nerve schwannomas should be included in the differential diagnosis in anterior cranial fossa space occupying lesions, and the approach should be designed taking into consideration, this rare entity.

Schwannomas are usually benign tumors, which arise from Schwann cells of central or peripheral nerves. They constitute 8% of all intracranial tumors and arise most commonly from the vestibular division of the 8 ^th^ cranial nerve. Other less common origins are the 5 ^th^ , 7 ^th^ , 9th, and 10 ^th^ cranial nerves. The occurrence of schwannoma not originating from cranial nerves is extremely rare, up to date. Only 66 cases have been reported 1 and most commonly they are located in the anterior cranial fossa. 2 3 4 Olfactory and optic nerves have no Schwann cells, which makes it theoretically impossible for schwannomas to arise in these locations, and arises a dilemma concerning the true pathogenesis of this tumor. 1 Here, we present a review of literature and a case of olfactory nerve schwannoma, which was accidently discovered, while evaluating a patient with head injury.

## Case Presentation

A 56-year-old male patient was presented to the emergency department, one day following a road traffic accident. Full history was obtained and thorough neurological examination was done, the patients Glasgow coma score was 13/15, he had right otorrhea, ecchymosis of both eyes, the right pupil was dilated fixed, and the left one was regular and reactive to light. Patient had history of old orthopedic instrumentation, 30 years ago.

CT (computed tomography) scan was obtained, which revealed pneumocephalus, and an incidental finding of a right frontal intra-axial mass with aggressive perilesional edema. Patient received conservative management, including dehydrating measures, antibiotics, and prophylactic antiepileptic. Complete investigations showed fracture maxilla and mandible.

**Fig. 1:**
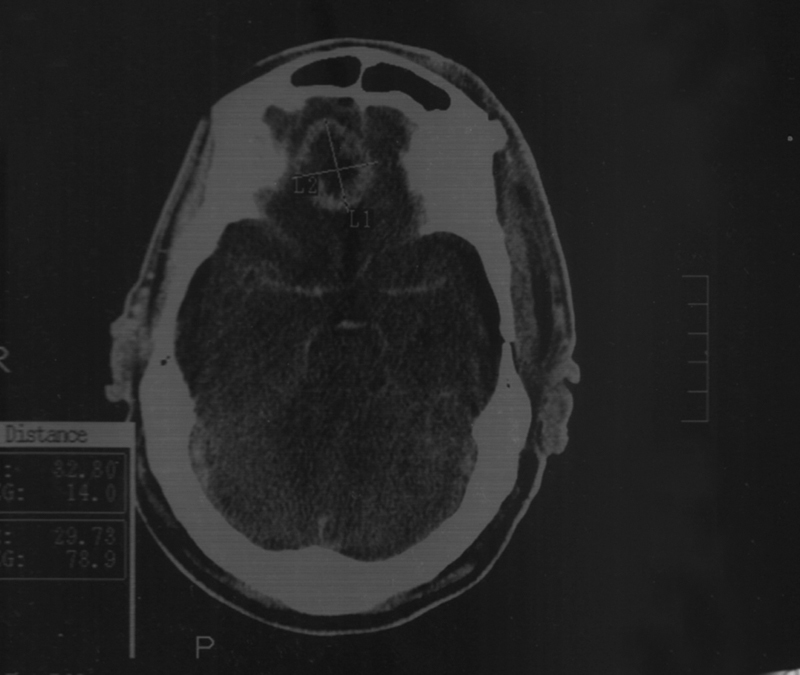
Postcontrast computed tomography (CT) scan, showing an intra-axial right frontal space-occupying lesion.

Two days after admission, patient regained consciousness and re-evaluation showed right sixth nerve palsy, right optic atrophy, and anosmia. We recommended magnetic resonance imaging (MRI) brain with contrast, but it was not possible due to the old fracture and instrumentation, so a CT scan with contrast was done, as shown in Fig. 1 , and revealed an intra-axial mass with perilesional edema, and a cystic component. Our differential diagnosis was a high-grade glioma, an abscess, or metastatic deposits. Metastatic workup including CT chest, pelvi-abdominal ultrasonography, and tumor markers including PSA, *α* -feto protein were all negative.

**Fig. 2:**
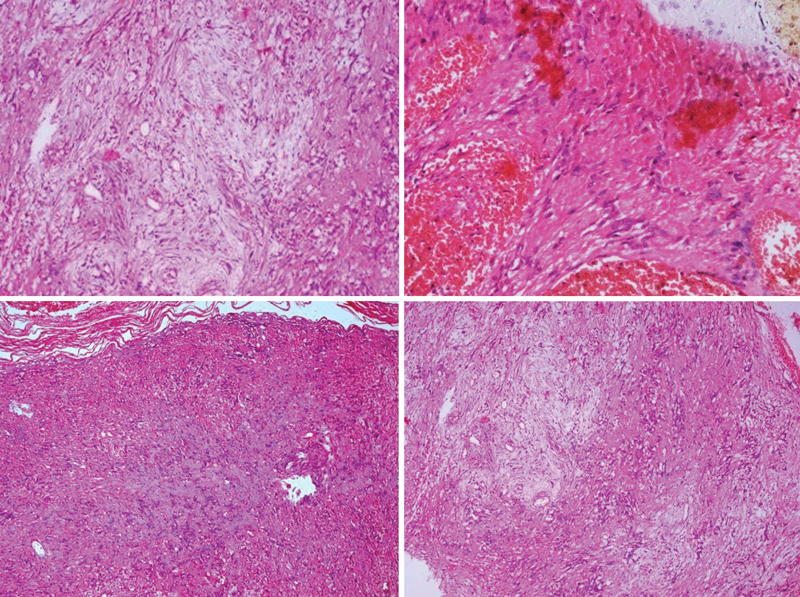
Histopathological examination, showing Antoni type-A cellular pattern.

Expecting the mass to be intra-axial a right frontal craniotomy with trans-cortical approach was decided. Intraoperatively, palpation of the brain surface revealed no underlying cystic lesion, neither did aspiration, using a brain needle came up with any fluid. We proceeded with the transcortical approach, where a well-defined basal intraparenchymal mass appeared. The mass was reddish, soft in consistency, and was excised completely at the end of the procedure. Postoperative CT scan is shown in Fig. 2 **.** We were not able to identify neither optic nerves, nor the olfactory, at the end of our transcortical approach.

**Fig. 3:**
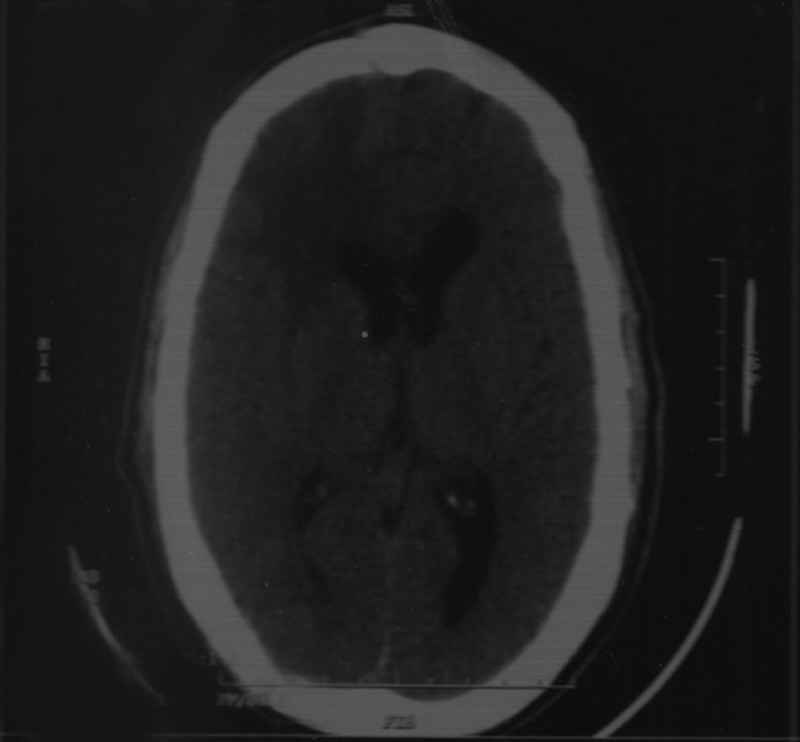
Postoperative noncontrast computed tomography (CT) scan, showing complete excision of the tumor.

Histopathological examination showed benign spindle-shaped cells, with elongated nuclei and fibrillary cytoplasm (Antoni-A pattern), and less cellular, loosely textured tumor areas, Fig. 3 .

Postoperatively, the patient was fully conscious, with improvement of his right sixth nerve palsy, but no improvement occurred in his smell, or right optic atrophy, right sixth nerve palsy, and anosmia.

## Discussion

Schwannomas of the anterior cranial fossa are extremely rare, only 66 cases have been reported in literature to date. 1 All reported cases were presented by headache, seizures, visual, or memory deficit. 1 2 In our case, although examination of the patient showed right anosmia, optic atrophy, and right sixth nerve palsy, the patient had never complained or gave any previous history apart from mild diminution of vision in the right eye.

The origin of this entity is still debatable, since the fact that olfactory nerve lacks Schwann cells and has only oligodendrocytes. 5 Existing theories suggest that these tumors arise from aberrant Schwann cells in the central nervous system (CNS), 6 another theory suggests its transformation from mesenchymal pial cells or migration of neural crest cells present inside the CNS, 7 both these theories may explain the origin of intraparenchymal schwannomas. 2 Some basic science publications came to findings that olfactory nerve has schwann cells, which arise from precursor cells, which are present in the olfactory epithelium, and this makes the fact about presence of schwann cells, or not in the olfactory nerve unsettled yet. 8 Other theories have been raised in a trial to explain the extra-axial or anterior skull base schwannomas, some authors posted that it may arise from the fila olfactoria, the embryonic terminal nerve, the nerve plexus of dural vessels, or from the ensheathing cells of the olfactory nerve and bulb. 6 9 10

Our approach to the tumor was through a right frontal transcortical craniotomy, although the tumor was completely excised and the patient came out with no neurological deficit, yet this was not the ideal approach to this lesion. The cause of this pitfall was absence of the MRI, which couldn't be done due to the previous orthopedic instrumentation and unfamiliarity with this rare entity of anterior fossa tumors. Most reported cases were done through a subfrontal approach, which offers the best surgical corridor to the tumor, 5 11 12 while some which had or had not a nasal sinus extension were done through endoscopy or endoscopy aided. 13 14 15 16 17 All these cases had MRI done and although the possibility of a schwannomas was not in the differential diagnosis, but the nature of the lesion being a basal one was taken into consideration.

We were not able to identify neither the optics nor the olfactory nerve, yet some of the other authors, who adopted a subfrontal approach couldn't identify the nerve also, but no conclusion could be made, whether it was due to the origin of the tumor or was involved in it or just a displacement. 2

## Conclusion

Despite being a rare type with 67 cases (including our case), reported worldwide up till now, anterior cranial fossa schwannoma should be included in the differential diagnosis of anterior cranial fossa lesions and further steps toward understanding the exact origin, pathology and pathogenesis of such tumor entities should be achieved.
